# Imbalances in Copper or Zinc Concentrations Trigger Further Trace Metal Dyshomeostasis in Amyloid-Beta Producing *Caenorhabditis elegans*

**DOI:** 10.3389/fnins.2021.755475

**Published:** 2021-10-11

**Authors:** Ada Metaxas

**Affiliations:** Princeton High School, Princeton, NJ, United States

**Keywords:** Alzheimer's, amyloid-beta, copper, zinc, trace metal, dyshomeostasis, *Caenorhabditis elegans*, imbalances

## Abstract

Alzheimer's Disease (AD), a progressive neurodegenerative disease characterized by the buildup of amyloid-beta (Aβ) plaques, is believed to be a disease of trace metal dyshomeostasis. Amyloid-beta is known to bind with high affinity to trace metals copper and zinc. This binding is believed to cause a conformational change in Aβ, transforming Aβ into a configuration more amenable to forming aggregations. Currently, the impact of Aβ-trace metal binding on trace metal homeostasis and the role of trace metals copper and zinc as deleterious or beneficial in AD remain elusive. Given that Alzheimer's Disease is the sixth leading cause of adult death in the U.S., elucidating the molecular interactions that characterize Alzheimer's Disease pathogenesis will allow for better treatment options. To that end, the model organism *C. elegans* is used in this study. *C. elegans*, a transparent nematode whose connectome has been fully established, is an amenable model to study AD phenomena using a multi-layered, interconnected approach. Aβ-producing and non-Aβ-producing *C. elegans* were individually supplemented with copper and zinc. On day 6 and day 9 after synchronization, the percent of worms paralyzed, concentration of copper, and concentration of zinc were measured in both groups of worms. This study demonstrates that dyshomeostasis of trace metals copper or zinc triggers further trace metal dyshomeostasis in Aβ-producing worms, while dyshomeostasis of copper or zinc triggers a return to equilibrium in non-Aβ-producing worms. This supports the characterization of Alzheimer's Disease as a disease of trace metal dyshomeostasis.

## Introduction

Alzheimer's Disease (AD) is the 6th leading cause of death in the U.S., with one in ten people age 65 or older having AD (Alzheimer's Association, 2021). As a progressive neurodegenerative disease, AD is characterized by extra-neuronal amyloid-beta plaques and intraneuronal tau neurofibrillary tangles which affect memory and cognition. Amyloid-beta plaques are aggregates of the amyloid-beta peptide (Aβ), a cleavage product of the amyloid precursor protein (APP). Buildup of Aβ causes neural death and neuroinflammation.

Neurodegenerative diseases such as Alzheimer's, Parkinson's, and Wilson's Diseases have been associated with metal dyshomeostasis, which often accompanies aging (Luo et al., 2011; Squitti, 2012; Singh et al., 2013). Metal dyshomeostasis occurs when metal levels increase or decrease beyond normal bounds. As important components of vitamins and enzymes, trace metals play a crucial role in neural and biochemical processes. When in homeostasis, these trace metals facilitate proper brain functioning and growth by protecting against reactive oxygen species (ROS), regulating gene expression, and activating enzymes. The dyshomeostasis of trace metals results in cellular damage and oxidative injury, induced by the formation of ROS (Grochowski et al., 2019).

Both trace metals copper and zinc play key roles in proper brain functioning. Copper is an essential trace element that plays a key role in energy production, free radicals scavenging, and neurotransmission (Singh et al., 2013). Zinc is another essential trace element that plays a key role in neurotransmission and redox regulation (Grochowski et al., 2019). Amyloid beta plaques have high affinity to trace metals copper and zinc and have thus been found to contain high concentrations of these trace metals (Bush et al., 1994; Atwood et al., 1998; Lovell et al., 1998; Sayre et al., 2000; Suh et al., 2000; Cherny et al., 2001; Dong et al., 2003; Miller et al., 2006; Mital et al., 2015; Ejaz et al., 2020). For instance, a 339% increase in Zn and a 466% increase in Cu were found in amyloid beta plaques of AD patients in comparison to healthy subjects (Leskovjan et al., 2009). The levels of copper and zinc in AD, however, remains controversial (Huang et al., 2000; Strausak et al., 2001; Cerpa et al., 2005; Kessler et al., 2005; Watt et al., 2010; Bagheri et al., 2018; Rana and Sharma, 2019). Some studies indicate copper deficiency in AD, suggesting a need for supplementation (Borchardt et al., 1999; Exley, 2006; Jiao and Yang, 2007; Kessler et al., 2008; Vural et al., 2010; Kaden et al., 2011; Exley et al., 2012; Xu et al., 2017), while others indicate copper excess in AD, suggesting a need for chelating agents (Cherny et al., 2001; Sparks et al., 2006; Hua et al., 2011; Luo et al., 2011; Ceccom et al., 2012; Eskici and Axelsen, 2012; Brewer, 2014; Squitti et al., 2014; Yu et al., 2015; Patel and Aschner, 2021). Similarly, some studies indicate zinc deficiency in AD (Kapaki et al., 1989; Molina et al., 1998; Brewer et al., 2010; Rivers-Auty et al., 2021), while others indicate zinc excess (Lovell et al., 1998; Religa et al., 2006; Bonda et al., 2011; Greenough et al., 2013; James et al., 2017). These conflicting findings could be partially due to differences in the brain regions in which copper and zinc were measured. With over 5 million Americans currently living with AD and nearly 14 million projected to be living with AD by 2050, better understanding the molecular mechanisms characterizing the involvement of copper and zinc dyshomeostasis in AD will allow for better treatment options and outcomes (Alzheimer's Association, 2021).

*Caenorhabditis elegans*, a non-parasitic nematode whose connectome has been fully established, is an advantageous model for studying the molecular mechanisms in Alzheimer's Disease (Caito et al., 2012). The nematode's simple nervous system and transparency allow for the study of the effects of AD on neuronal pathways and function. Roughly 38% of worm genes have a human ortholog, such as APP and tau, making *C. elegans* an excellent *in vivo* model for the study of AD (Shaye and Greenwald, 2011). Since the toxic Aβ42-peptide is expressed in muscle cells in *C. elegans* strain CL2006, Aβ aggregations result in the paralysis of *C. elegans*, thus allowing the extent of Aβ aggregation in response to different treatments to be viewed macroscopically (Saharia et al., 2016).

Given that molecular mechanisms characterizing the interaction between copper, zinc, and amyloid-beta remain elusive, the present study aims to elucidate whether the dyshomeostasis of one trace metal induces the dyshomeostasis of other trace metals and of amyloid-beta in Alzheimer's Disease. It is hypothesized that increases in amyloid-beta aggregations are part of a failed protective homeostatic mechanism to bind excess trace metals copper and zinc. The present study newly shows that dyshomeostasis of trace metals copper or zinc triggers further trace metal dyshomeostasis in Aβ-producing worms while dyshomeostasis of copper or zinc triggers a return to equilibrium in non-Aβ-producing worms.

## Materials and Methods

### Nematode Strains and Maintenance

*Caenorhabditis elegans* strains were received from the Caenorhabditis Genetics Center (CGC). The transgenic *C. elegans* strain CL2006, which expresses human Aβ_1−42_ in body-wall muscle cells, is characterized by progressive, adult-onset paralysis and a roller phenotype (Link, 1995). The *C. elegans* strain N2 represents the wild type. During two independent trials, worm strains were synchronized according to the following procedure: Adult hermaphrodite worms were transferred to fresh plates and allowed to lay eggs for 2–4 h. After removal of the adult parental worms, the synchronized progeny were allowed to reach adulthood, then later scored for paralysis (Fonte et al., 2002). The worms were propagated at 20**°**C on Nematode Growth Media (NGM) plates seeded with the bacterial strain OP50 and supplemented with either copper or zinc (Brenner, 1974).

### Supplementation With Copper and Zinc

CuCl_2_ was used to supplement the worms with copper. CuCl_2_ stock solution was diluted into a live E. coli OP50 suspension, reaching a final concentration of 150 μM, and was placed on the surface of the NGM plates. Once the worms reached adulthood (day 3), a group of synchronized CL2006 worms and a group of synchronized N2 worms were placed on the copper-supplemented plates. ZnSO_4_ was used to supplement the worms with zinc. ZnSO_4_ stock solution was diluted into a live E. coli OP50 suspension, reaching a final concentration of 500 μM, and was placed on the surface of the NGM plates. Once the worms reached adulthood, a group of synchronized CL2006 worms and a group of synchronized N2 worms were placed on these zinc-supplemented plates.

### Paralysis Assay

On days 6 and 9^1^ after synchronization, 20 worms from the copper-supplemented and zinc-supplemented CL2006 and N2 groups were tested for paralysis. Paralysis indicates the extent of Aβ-aggregation development. The worms were tested for paralysis by tapping their noses with a platinum wire pick. Worms that moved their noses but failed to move their bodies were scored as “paralyzed” (Luo et al., 2011).

### Lysis Procedure

On days 6 and 9, thirty worms from each of the four groups: (1) copper-supplemented CL2006, (2) zinc-supplemented CL2006, (3) copper-supplemented N2, (4) zinc-supplemented N2, were lysed in preparation for copper and zinc colorimetric assays. The following procedure is especially useful for dauer larvae lysis. Worms were spun in a centrifuge at 4,000 rpm for 1 min to a pellet. The supernatant was removed, and the pellet was washed in 1.5 mL of ice cold L15 buffer. The worms were centrifuged, and the supernatant was removed once again. Twenty-five microliters of the pellet was pipetted onto a glass slide. A 50 mm glass coverslip was added on top, and pressure was applied to the coverslip using a pipette. When viewed under a microscope, head disruption head could be visualized as pressure was applied with the pipette tip. Pressure continued to be applied until most of the worms were exploded. The contents on the coverslip and slide were washed off with 1 ml of cold L15 into a test tube. Finally, this L15-cell solution was pipetted vigorously 25 times to ensure the *C. elegans* were completely lysed.

### Copper and Zinc Colorimetric Assays

To quantify the amount of copper in the *C. elegans* on days 6 and 9, a copper colorimetric assay (Elabscience) was applied to the lysed *C. elegans* solution. Similarly, to quantify the amount of zinc in the *C. elegans* on days 6 and 9, a zinc colorimetric assay (Elabscience) was applied to the lysed *C. elegans* solution. Once the standard wells were created for both assays, the percent transmittance of the standards and test groups was measured using a colorimeter. The percent transmittance was converted to ion content (μmol/L) as specified by the Elabscience assays.

### Statistical Analysis

All values were expressed as mean ± SEM. Statistical analysis involving two groups was conducted using a *t*-test. Statistical analysis involving more than two groups was conducted using a one-way analysis of variance (ANOVA) followed by a *post-hoc* analysis using Tukey test. The differences were considered to be significant at *p* < 0.05.

## Results

To elucidate the differences in trace metal homeostasis maintenance in amyloid-beta producing *C. elegans* compared to non-amyloid-beta producing *C. elegans*, both strains of *C. elegans* were supplemented with copper and zinc individually.

### Zinc Concentration Changes in Response to Copper Supplementation

**Figure 1:**
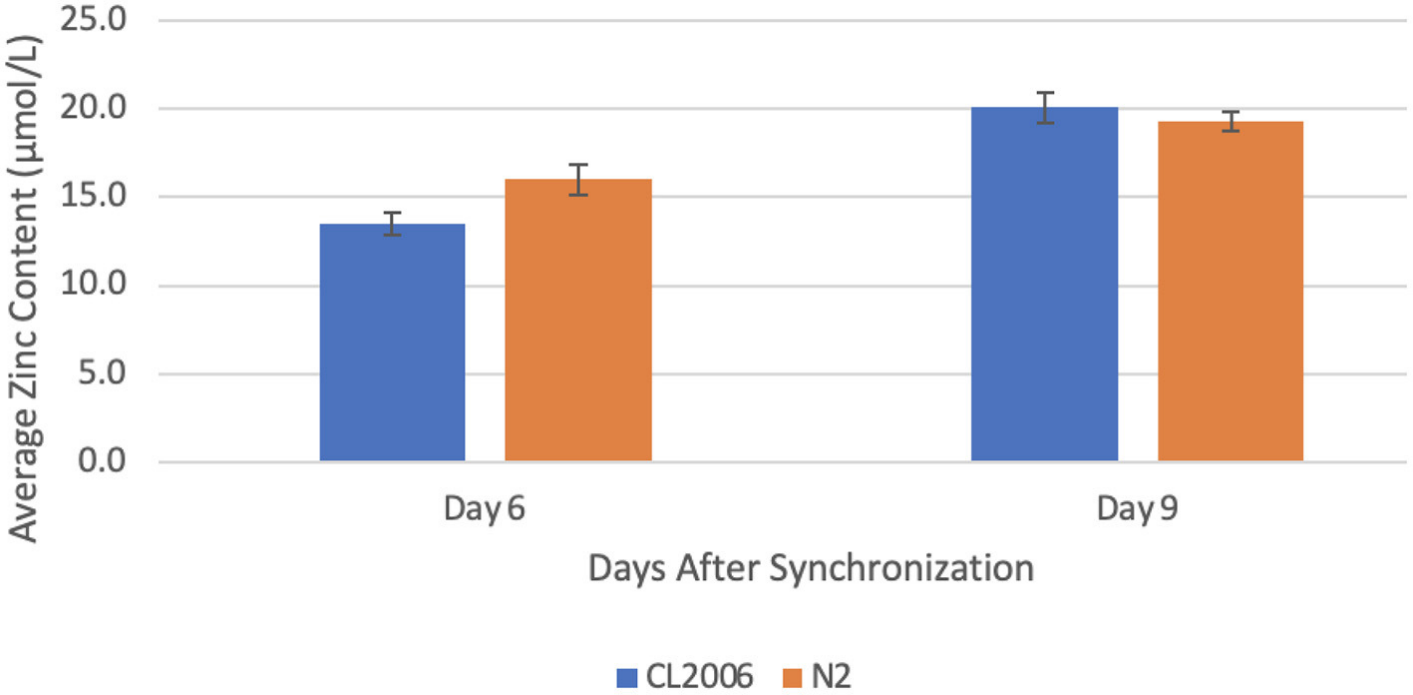
Average zinc content in *C. elegans* supplemented with copper. When amyloid-beta producing *C. elegans* (CL2006) were supplemented with copper, a statistically significant (*p* = 0.013) increase in the average zinc content occurred from day 6 (13.5 ± 0.6 μmol/L) to day 9 (20.1 ± 0.9 μmol/L). Similarly, when non-amyloid-beta producing *C. elegans* (N2) were supplemented with copper, a statistically significant (*p* = 0.041) increase in the average zinc content occurred from day 6 (16.0 ± 0.9 μmol/L) to day 9 (19.3 ± 0.6 μmol/L). Values are mean ± SEM and are representative of 2 experiments where 30 *C. elegans* were analyzed at each time point.

When Aβ-producing *C. elegans* were supplemented with copper, the zinc concentration increased significantly (*p* = 0.013) from day 6 (13.5 ± 0.6 μmol/L) to day 9 (20.1 ± 0.9 μmol/L). Likewise, when wild-type worms were supplemented with copper, the zinc concentration increased significantly (*p* = 0.041) from day 6 (16.0 ± 0.9 μmol/L) to day 9 (19.3 ± 0.6 μmol/L, Figure 1). Additionally, the percent change in zinc content from day 6 to day 9 in Aβ-producing C. elegans (49% increase) was more than double the percent change in wild-type *C. elegans* (21% increase). This indicates that a high copper concentration results in a larger change in the zinc concentration in Aβ-producing *C. elegans* compared to non-Aβ-producing *C. elegans*.

### Copper Concentration Changes in Response to Zinc Supplementation

**Figure 2:**
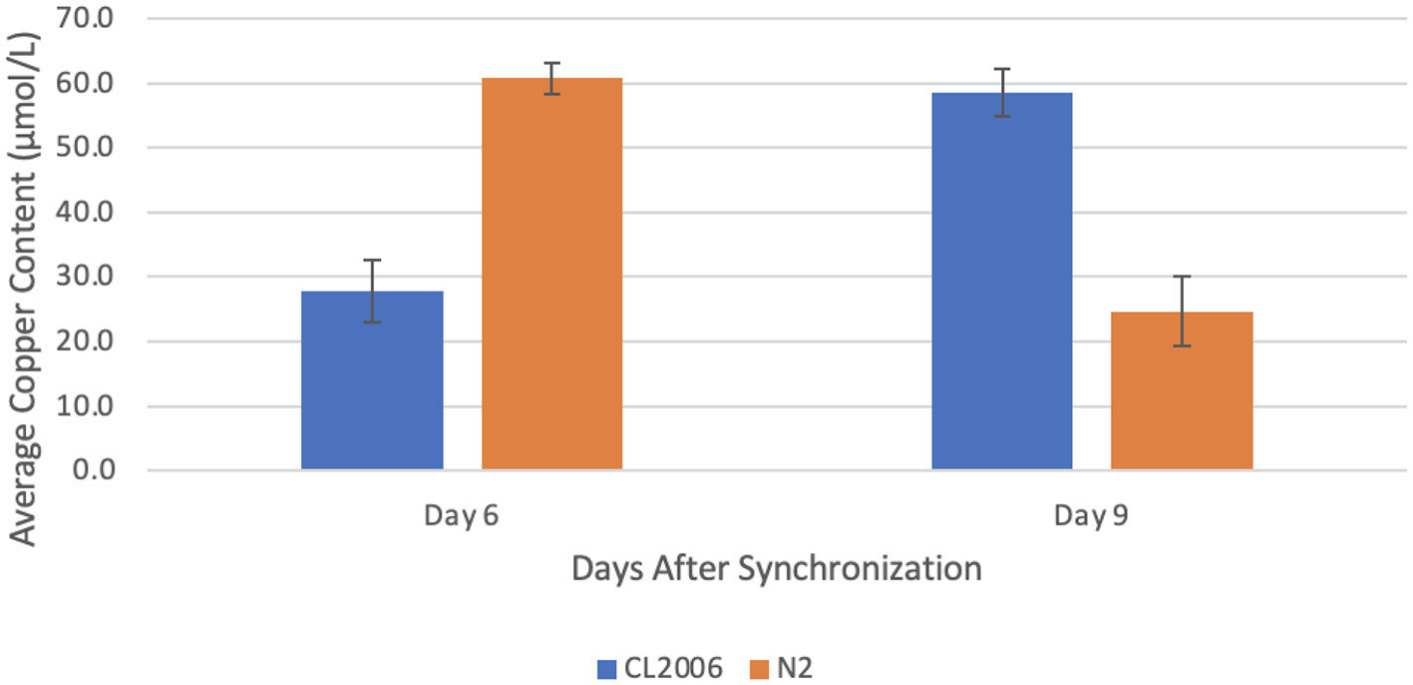
Average copper content in *C. elegans* supplemented with zinc. When amyloid-beta producing *C. elegans* (CL2006) were supplemented with zinc, a statistically significant (*p* = 0.022) increase in the average copper content occurred from day 6 (27.8 ± 4.9 μmol/L) to day 9 (58.6 ± 3.7 μmol/L). In contrast, when non-amyloid-beta producing *C. elegans* (N2) were supplemented with zinc, a statistically significant (*p* = 0.012) decrease in the average copper content occurred from day 6 (60.8 ± 2.4 μmol/L) to day 9 (24.7 ± 5.4 μmol/L). Values are mean ± SEM and are representative of 2 experiments where 30 *C. elegans* were analyzed at each time point.

When Aβ-producing *C. elegans* were supplemented with zinc, the copper concentration increased significantly (*p* = 0.022) from day 6 (27.8 ± 4.9 μmol/L) to day 9 (58.6 ± 3.7 μmol/L). In contrast, when wild-type *C. elegans* were supplemented with zinc, the copper concentration decreased significantly (*p* = 0.012) from day 6 (60.8 ± 2.4 μmol/L) to day 9 (24.7 ± 5.4 μmol/L, Figure 2). In fact, the copper content on day 6 in mutant *C. elegans* was roughly equivalent to the copper content on day 9 in wild-type *C. elegans* (*p* = 0.9). Similarly, the copper content on day 9 in mutant *C. elegans* was roughly equivalent to the copper content on day 6 in wild-type *C. elegans* (*p* = 0.9). This indicates that a high zinc concentration through supplementation results in an increase in the copper content of Aβ-producing *C. elegans* that is roughly equal in magnitude to the decrease in copper content in non-Aβ-producing *C. elegans*.

### Effect of Copper and Zinc Dyshomeostasis on Aβ Aggregations

**Figure 3:**
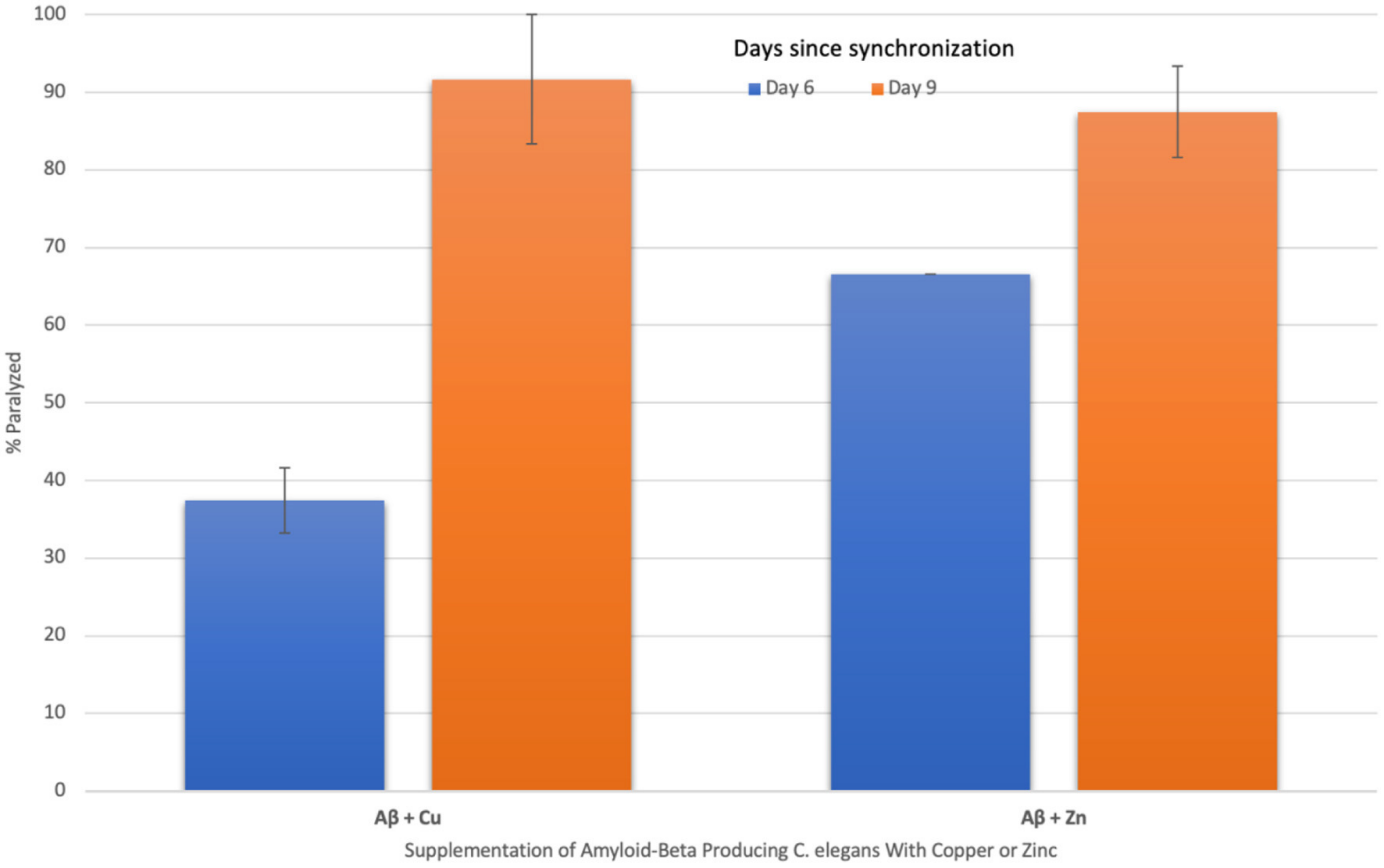
The effect of supplementing amyloid-beta producing *C. elegans* with copper and zinc on percent paralyzed over time. When amyloid-beta producing *C. elegans* were supplemented with copper, a statistically significant (*p* = 0.014) increase in % paralyzed occurred from day 6 (37 ± 4) to day 9 (92 ± 8). When supplemented with zinc, a statistically significant (*p* = 0.019) increase in % paralyzed also occurred from day 6 (67 ± 0) to day 9 (87 ± 4). The change in percent paralyzed is larger for the copper-supplemented group compared to the zinc-supplemented group. Values are mean ± SEM and are representative of 2 experiments where 20 *C. elegans* were analyzed at each time point.

To characterize the effect of imbalances in such trace metals on the extent of Aβ aggregations, the percent of worms paralyzed was measured in Aβ-producing *C. elegans* supplemented with copper or zinc. The percent of worms paralyzed significantly increased in both copper-supplemented mutant worms (*p* = 0.0142) from day 6 (37 ± 4%) to day 9 (92 ± 8%) and zinc-supplemented mutant worms (*p* = 0.0187) from day 6 (67 ± 0%) to day 9 (87 ± 4%), as shown in Figure 3. The change in percent paralyzed from day 6 to day 9 was larger for the copper-supplemented group (145% increase) compared to the zinc-supplemented group (31% increase). Also, the percent paralyzed was significantly higher on day 6 for the zinc-supplemented group compared to the copper-supplemented group (*p* = 0.0098), while there was no significant difference in percent of the worms paralyzed between the two groups on day 9 (*p* = 0.3483). Overall, high concentrations of both copper and zinc are positively correlated with increases in the percent of worms paralyzed.

## Discussion

Aging has been found to trigger copper and zinc dyshomeostasis (Myhre et al., 2013; McCord and Aizenman, 2014; Nuttall and Oteiza, 2014; Malavolta et al., 2015). While trace metals copper and zinc are crucial for normal functioning, excess copper, and zinc are highly damaging to proteins. Excess copper and zinc are known to bind with high affinity to Aβ, resulting in visible precipitation into an aggregated form (Bush et al., 1994; Huang et al., 1997, 2000; Atwood et al., 2000; Kumar et al., 2016; Bagheri et al., 2018; Barykin et al., 2018). Therefore, it is of particular interest to determine how changes in the homeostasis of a given trace metal influence the homeostasis of other trace metals and the aggregation state of Aβ.

The present study has found that in Aβ-producing *C. elegans*, imbalances in trace metals copper or zinc trigger further trace metal dyshomeostasis. When supplemented with copper, zinc levels increase significantly and when supplemented with zinc, copper levels increase significantly. Thus, an imbalance in either trace metal causes a cascading effect resulting in further imbalances. This triggering of further trace metal dyshomeostasis might explain why the percent of worms paralyzed, which correlates to Aβ-aggregation levels, significantly increases in both copper and zinc supplemented mutant worms from day 6 to day 9.

However, in wild-type worms, dyshomeostasis of copper or zinc ultimately triggers a return to equilibrium. When copper levels increase through supplementation, zinc levels correspondingly increase from day 6 to day 9. In contrast, when zinc levels increase through supplementation, copper levels decrease from day 6 to day 9. Therefore, it is possible that through a negative feedback mechanism loop, an increase in copper triggers an increase in zinc which ultimately causes a decrease in copper and a return to equilibrium.

Increases in the concentration of zinc or copper, through supplementation, both result in increases in the percent of worms paralyzed, reflecting higher Aβ-aggregation levels, from day 6 to day 9. Given that trace metal levels naturally increase to a degree during the aging process, it is possible that in certain populations more prone to developing amyloidogenic diseases, trace metal levels dramatically increase during aging. Since Aβ avidly binds to trace metals copper and zinc, it is possible that when trace metal levels increase during the aging process, Aβ levels increase in an effort to bind excess copper and zinc (Squitti et al., 2021). The binding of trace metals such as copper and zinc to Aβ is known to trigger an Aβ conformational shape change (Barykin et al., 2018; Kim et al., 2018; De Benedictis et al., 2019), thus transforming Aβ into a configuration more amenable to forming aggregations.

While both copper and zinc dyshomeostasis result in an increase in the percent of worms paralyzed over time, copper might have a stronger effect on the percent of worms paralyzed, reflecting Aβ aggregations, compared to zinc. The zinc supplementation concentration (500 μM ZnSO_4_) was over three times higher than the copper supplementation concentration (150 μM CuCl_2_); however, the percent of worms paralyzed on day 6 in the zinc supplemented group was only about 1.8 times higher than the copper supplemented group. The zinc supplementation (Kumar et al., 2016) and copper supplementation (Minniti et al., 2009) were chosen based on previous publications that found considerable changes in amyloid-beta aggregations, but did not measure whether dyshomeostasis of one trace metal triggers dyshomeostasis of other trace metals. Additionally, despite the higher zinc supplementation, there was no significant difference in the percent of worms paralyzed by day 9 when comparing the zinc supplemented group and the copper supplemented group. While this could simply be due to natural age-related Aβ aggregation development caused by the inserted Aβ gene as the mutant *C. elegans* approach the end of their lifespan, more trials would be needed to better understand if there is a significant difference between the effect of copper vs. zinc on Aβ aggregations. It would be particularly useful to measure the percent of worms paralyzed when supplementing with the same concentration of CuCl_2_ and ZnSO_4_ in the future. Future work also includes measuring the levels of copper and zinc in Aβ-producing and non-Aβ-producing *C. elegans* without any supplementation to determine the copper and zinc homeostatic ranges.

Overall, the novelty of this study is the experimental demonstration that dyshomeostasis of trace metals copper or zinc triggers further trace metal dyshomeostasis in Aβ-producing worms, while dyshomeostasis of copper or zinc triggers a return to equilibrium in non-Aβ-producing worms. Future directions include determining how increases in amyloid-beta aggregations might be part of a failed protective homeostatic mechanism to bind excess trace metals copper and zinc. Additional future directions will include elucidating the mediating factors that facilitate Aβ-trace metal binding and testing the effects of trace metal chelators on Aβ levels.

## Data Availability Statement

The original contributions presented in the study are included in the article/supplementary material, further inquiries can be directed to the corresponding author/s.

## Author Contributions

The author confirms being the sole contributor of this work and has approved it for publication.

## Funding

This research was supported by crowdfunding through the website experiment.com.

## Conflict of Interest

The author declares that the research was conducted in the absence of any commercial or financial relationships that could be construed as a potential conflict of interest.

## Publisher's Note

All claims expressed in this article are solely those of the authors and do not necessarily represent those of their affiliated organizations, or those of the publisher, the editors and the reviewers. Any product that may be evaluated in this article, or claim that may be made by its manufacturer, is not guaranteed or endorsed by the publisher.

## Footnotes

^1^Day 6 was chosen to ensure that the effects of supplementation, which occurred when worms reached adulthood on day 3, will be significant. Day 9 was chosen as the last date of data collection to ensure that dead worms were not mistaken for paralyzed worms.

## References

[B1] Alzheimer's Association (2021). Alzheimer's Disease Facts and Figures. Alzheimer's Association. Available online at: https://www.alz.org/alzheimers-dementia/facts-figures (accessed August 8, 2021).

[B2] Atwood C. S. Moir R. D. Huang X. Scarpa R. C. Bacarra N. M. Romano D. M.. (1998). Dramatic aggregation of Alzheimer abeta by Cu(II) is induced by conditions representing physiological acidosis. J. Biol. Chem. 273, 12817–12826. 10.1074/jbc.273.21.12817 9582309

[B3] Atwood C. S. Scarpa R. C. Huang X. Moir R. D. Jones W. D. Fairlie D. P.. (2000). Characterization of copper interactions with alzheimer amyloid beta peptides: identification of an attomolar-affinity copper binding site on amyloid beta1-42. J. Neurochem. 75, 1219–1233. 10.1046/j.1471-4159.2000.0751219.x 10936205

[B4] Bagheri S. Squitti R. Haertlé T. Siotto M. Saboury A. A. (2018). Role of copper in the onset of Alzheimer's disease compared to other metals. Front. Aging Neurosci. 9:446. 10.3389/fnagi.2017.00446 29472855 PMC5810277

[B5] Barykin E. P. Petrushanko I. Y. Kozin S. A. Telegin G. B. Chernov A. S. Lopina O. D.. (2018). Phosphorylation of the amyloid-beta peptide inhibits zinc-dependent aggregation, prevents Na,K-ATPase inhibition, and reduces cerebral plaque deposition. Front. Mol. Neurosci. 11:302. 10.3389/fnmol.2018.00302 30210292 PMC6123382

[B6] Bonda D. J. Lee H. G. Blair J. A. Zhu X. Perry G. Smith M. A. (2011). Role of metal dyshomeostasis in Alzheimer's disease. Metallomic 3, 267–270. 10.1039/c0mt00074d 21298161 PMC3117398

[B7] Borchardt T. Camakaris J. Cappai R. Masters C. L. Beyreuther K. Multhaup G. (1999). Copper inhibits beta-amyloid production and stimulates the non-amyloidogenic pathway of amyloid-precursor-protein secretion. Biochem. J. 344(Pt 2), 461–467. 10567229 PMC1220664

[B8] Brenner S. (1974). The genetics of *Caenorhabditis elegans*. Genetics 77, 71–94. 4366476 10.1093/genetics/77.1.71 PMC1213120

[B9] Brewer G. J. (2014). Alzheimer's disease causation by copper toxicity and treatment with zinc. Front. Aging Neurosci. 6:92. 10.3389/fnagi.2014.00092 24860501 PMC4030141

[B10] Brewer G. J. Kanzer S. H. Zimmerman E. A. Molho E. S. Celmins D. F. Heckman S. M.. (2010). Subclinical zinc deficiency in Alzheimer's disease and Parkinson's disease. Am. J. Alzheimers Dis. Other Dement. 25, 572–575. 10.1177/1533317510382283 20841345 PMC10845304

[B11] Bush A. I. Pettingell W. H. Multhaup G. d Paradis M. Vonsattel J. P. Gusella J. F.. (1994). Rapid induction of Alzheimer A beta amyloid formation by zinc. Science 265, 1464–1467. 10.1126/science.8073293 8073293

[B12] Caito S. Fretham S. Martinez-Finley E. Chakraborty S. Avila D. Chen P.. (2012). Genome-wide analyses of metal responsive genes in *Caenorhabditis elegans*. Front. Genet. 3:52. 10.3389/fgene.2012.00052 22514555 PMC3322339

[B13] Ceccom J. Coslédan F. Halley H. Francès B. Lassalle J. M. Meunier B. (2012). Copper chelator induced efficient episodic memory recovery in a non-transgenic Alzheimer's mouse model. PLoS ONE 7:e43105. 10.1371/journal.pone.0043105 22927947 PMC3424235

[B14] Cerpa W. Varela-Nallar L. Reyes A. E. Minniti A. N. Inestrosa N. C. (2005). Is there a role for copper in neurodegenerative diseases? Mol. Aspects Med. 26, 405–420. 10.1016/j.mam.2005.07.011 16112188

[B15] Cherny R. A. Atwood C. S. Xilinas M. E. Gray D. N. Jones W. D. McLean C. A.. (2001). Treatment with a copper-zinc chelator markedly and rapidly inhibits beta-amyloid accumulation in Alzheimer's disease transgenic mice. Neuron 30, 665–676. 10.1016/s0896-6273(01)00317-8 11430801

[B16] De Benedictis C. A. Vilella A. Grabrucker A. M. (2019). The role of trace metals in Alzheimer's disease, in Alzheimer's Disease, ed T. Wisniewski (Brisbane, AU: Codon Publications), 85–106. 10.15586/alzheimersdisease.2019.ch6 31895516

[B17] Dong J. Atwood C. S. Anderson V. E. Siedlak S. L. Smith M. A. Perry G.. (2003). Metal binding and oxidation of amyloid-beta within isolated senile plaque cores: Raman microscopic evidence. Biochemistry 42, 2768–2773. 10.1021/bi0272151 12627941

[B18] Ejaz H. W. Wang W. Lang M. (2020). Copper toxicity links to pathogenesis of Alzheimer's disease and therapeutics approaches. Int. J. Mol. Sci. 21:7660. 10.3390/ijms21207660 33081348 PMC7589751

[B19] Eskici G. Axelsen P. H. (2012). Copper and oxidative stress in the pathogenesis of Alzheimer's disease. Biochemistry 51, 6289–6311. 10.1021/bi3006169 22708607

[B20] Exley C. (2006). Aluminium and iron, but neither copper nor zinc, are key to the precipitation of beta-sheets of Abeta_{42} in senile plaque cores in Alzheimer's disease. J. Alzheimers Dis. 10, 173–177. 10.3233/jad-2006-102-305 17119286

[B21] Exley C. House E. Polwart A. Esiri M. M. (2012). Brain burdens of aluminum, iron, and copper and their relationships with amyloid-β pathology in 60 human brains. J. Alzheimers Dis. 31, 725–730. 10.3233/JAD-2012-120766 22699848

[B22] Fonte V. Kapulkin W. J. Taft A. Fluet A. Friedman D. Link C. D. (2002). Interaction of intracellular beta amyloid peptide with chaperone proteins. Proc. Natl. Acad. Sci. U.S.A. 99, 9439–9444. 10.1073/pnas.152313999 12089340 PMC123159

[B23] Greenough M. A. Camakaris J. Bush A. I. (2013). Metal dyshomeostasis and oxidative stress in Alzheimer's disease. Neurochem. Int. 62, 540–555. 10.1016/j.neuint.2012.08.014 22982299

[B24] Grochowski C. Blicharska E. Krukow P. Jonak K. Maciejewski M. Szczepanek D.. (2019). Analysis of trace elements in human brain: its aim, methods, and concentration levels. Front. Chem. 7:115. 10.3389/fchem.2019.00115 30891444 PMC6411644

[B25] Hua H. Münter L. Harmeier A. Georgiev O. Multhaup G. Schaffner W. (2011). Toxicity of Alzheimer's disease-associated Aβ peptide is ameliorated in a *Drosophila* model by tight control of zinc and copper availability. Biol. Chem. 392, 919–926. 10.1515/BC.2011.084 21801085

[B26] Huang X. Atwood C. S. Moir R. D. Hartshorn M. A. Vonsattel J. P. Tanzi R. E.. (1997). Zinc-induced Alzheimer's Abeta1-40 aggregation is mediated by conformational factors. J. Biol. Chem. 272, 26464–26470. 10.1074/jbc.272.42.26464 9334223

[B27] Huang X. Cuajungco M. P. Atwood C. S. Moir R. D. Tanzi R. E. Bush A. I. (2000). Alzheimer's disease, beta-amyloid protein and zinc. J. Nutr. 130, 1488S−1492S. 10.1093/jn/130.5.1488S 10801964

[B28] James S. A. Churches Q. I. de Jonge M. D. Birchall I. E. Streltsov V. McColl G.. (2017). Iron, copper, and zinc concentration in Aβ plaques in the APP/PS1 mouse model of Alzheimer's disease correlates with metal levels in the surrounding neuropil. ACS Chem. Neurosci. 8, 629–637. 10.1021/acschemneuro.6b00362 27958708

[B29] Jiao Y. Yang P. (2007). Mechanism of copper(II) inhibiting Alzheimer's amyloid beta-peptide from aggregation: a molecular dynamics investigation. J. Phys. Chem. B 111, 7646–7655. 10.1021/jp0673359 17564430

[B30] Kaden D. Bush A. I. Danzeisen R. Bayer T. A. Multhaup G. (2011). Disturbed copper bioavailability in Alzheimer's disease. Int. J. Alzheimers Dis. 2011:345614. 10.4061/2011/345614 22145082 PMC3227474

[B31] Kapaki E. Segditsa J. Papageorgiou C. (1989). Zinc, copper and magnesium concentration in serum and CSF of patients with neurological disorders. Acta Neurol. Scand. 79, 373–378. 10.1111/j.1600-0404.1989.tb03803.x 2545071

[B32] Kessler H. Pajonk F. G. Bach D. Schneider-Axmann T. Falkai P. Herrmann W.. (2008). Effect of copper intake on CSF parameters in patients with mild Alzheimer's disease: a pilot phase 2 clinical trial. J. Neural Transm. 115, 1651–1659. 10.1007/s00702-008-0136-2 18972062

[B33] Kessler H. Pajonk F. G. Supprian T. Falkai P. Multhaup G. Bayer T. A. (2005). The role of copper in the importance of copper in the pathophysiology of Alzheimer's disease. Neurologist 76, 581–585. 10.1007/s00115-004-1849-6 15905983

[B34] Kim A. C. Lim S. Kim Y. K. (2018). Metal ion effects on Aβ and tau aggregation. Int. J. Mol. Sci. 19:128. 10.3390/ijms19010128 29301328 PMC5796077

[B35] Kumar J. Barhydt T. Awasthi A. Lithgow G. J. Killilea D. W. Kapahi P. (2016). Zinc levels modulate lifespan through multiple longevity pathways in *Caenorhabditis elegans*. PLoS ONE 11:e0153513. 10.1371/journal.pone.0153513 27078872 PMC4831763

[B36] Leskovjan A. C. Lanzirotti A. Miller L. M. (2009). Amyloid plaques in PSAPP mice bind less metal than plaques in human Alzheimer's disease. Neuroimage 47, 1215–1220. 10.1016/j.neuroimage.2009.05.063 19481608 PMC2746706

[B37] Link C. D. (1995). Expression of human beta-amyloid peptide in transgenic *Caenorhabditis elegans*. Proc. Natl. Acad. Sci. U.S.A. 92, 9368–9372. 10.1073/pnas.92.20.9368 7568134 PMC40986

[B38] Lovell M. A. Robertson J. D. Teesdale W. J. Campbell J. L. Markesbery W. R. (1998). Copper, iron and zinc in Alzheimer's disease senile plaques. J. Neurol. Sci. 158, 47–52. 10.1016/s0022-510x(98)00092-6 9667777

[B39] Luo Y. Zhang J. Liu N. Luo Y. Zhao B. (2011). Copper ions influence the toxicity of β-amyloid(1-42) in a concentration-dependent manner in a *Caenorhabditis elegans* model of Alzheimer's disease. Sci. China Life Sci. 54, 527–534. 10.1007/s11427-011-4180-z 21706413

[B40] Malavolta M. Piacenza F. Basso A. Giacconi R. Costarelli L. Mocchegiani E. (2015). Serum copper to zinc ratio: relationship with aging and health status. Mech. Ageing Dev. 151, 93–100. 10.1016/j.mad.2015.01.004 25660061

[B41] McCord M. C. Aizenman E. (2014). The role of intracellular zinc release in aging, oxidative stress, and Alzheimer's disease. Front. Aging Neurosci. 6:77. 10.3389/fnagi.2014.00077 24860495 PMC4028997

[B42] Miller L. M. Wang Q. Telivala T. P. Smith R. J. Lanzirotti A. Miklossy J. (2006). Synchrotron-based infrared and X-ray imaging shows focalized accumulation of Cu and Zn co-localized with beta-amyloid deposits in Alzheimer's disease. J. Struct. Biol. 155, 30–37. 10.1016/j.jsb.2005.09.004 16325427

[B43] Minniti A. N. Rebolledo D. L. Grez P. M. Fadic R. Aldunate R. Volitakis I.. (2009). Intracellular amyloid formation in muscle cells of Abeta-transgenic *Caenorhabditis elegans*: determinants and physiological role in copper detoxification. Mol. Neurodegener. 4:2. 10.1186/1750-1326-4-2 19126228 PMC2632641

[B44] Mital M. Wezynfeld N. E. Fraczyk T. Wiloch M. Z. Wawrzyniak U. E. Bonna A.. (2015). A functional role for Aβ in metal homeostasis? N-truncation and high-affinity copper binding. Angew. Chem. 54, 10460–10464. 10.1002/anie.201502644 26178596

[B45] Molina J. A. Jiménez-Jiménez F. J. Aguilar M. V. Meseguer I. Mateos-Vega C. J. González-Muñoz M. J.. (1998). Cerebrospinal fluid levels of transition metals in patients with Alzheimer's disease. J. Neural Transm. 105, 479–488. 10.1007/s007020050071 9720975

[B46] Myhre O. Utkilen H. Duale N. Brunborg G. Hofer T. (2013). Metal dyshomeostasis and inflammation in Alzheimer's and Parkinson's diseases: possible impact of environmental exposures. Oxid. Med. Cell. Longev. 2013:726954. 10.1155/2013/726954 23710288 PMC3654362

[B47] Nuttall J. R. Oteiza P. I. (2014). Zinc and the aging brain. Genes Nutr. 9:379. 10.1007/s12263-013-0379-x 24366781 PMC3896632

[B48] Patel R. Aschner M. (2021). Commonalities between copper neurotoxicity and Alzheimer's disease. Toxics 9:4. 10.3390/toxics9010004 33430181 PMC7825595

[B49] Rana M. Sharma A. K. (2019). Cu and Zn interactions with Aβ peptides: consequence of coordination on aggregation and formation of neurotoxic soluble Aβ oligomers. Metallomics 11, 64–84. 10.1039/c8mt00203g 30234208

[B50] Religa D. Strozyk D. Cherny R. A. Volitakis I. Haroutunian V. Winblad B.. (2006). Elevated cortical zinc in Alzheimer disease. Neurology 67, 69–75. 10.1212/01.wnl.0000223644.08653.b5 16832080

[B51] Rivers-Auty J. Tapia V. S. White C. S. Daniels M. Drinkall S. Kennedy P. T.. (2021). Zinc status alters Alzheimer's disease progression through NLRP3-dependent inflammation. J. Neurosci. 41, 3025–3038. 10.1523/JNEUROSCI.1980-20.2020 33597269 PMC8018890

[B52] Saharia K. Kumar R. Gupta K. Mishra S. Subramaniam J. (2016). A novel way of amelioration of amyloid beta induced toxicity in *Caenorhabditis elegans*. Ann. Neurosci. 23, 149–154. 10.1159/000449180 27721583 PMC5043337

[B53] Sayre L. M. Perry G. Harris P. L. Liu Y. Schubert K. A. Smith M. A. (2000). *In situ* oxidative catalysis by neurofibrillary tangles and senile plaques in Alzheimer's disease: a central role for bound transition metals. J. Neurochem. 74, 270–279. 10.1046/j.1471-4159.2000.0740270.x 10617129

[B54] Shaye D. D. Greenwald I. (2011). OrthoList: a compendium of *C. elegans* genes with human orthologs. PLoS ONE 6:e20085. 10.1371/journal.pone.0020085 21647448 PMC3102077

[B55] Singh I. Sagare A. P. Coma M. Perlmutter D. Gelein R. Bell R. D.. (2013). Low levels of copper disrupt brain amyloid-β homeostasis by altering its production and clearance. Proc. Natl. Acad. Sci. U.S.A. 110, 14771–14776. 10.1073/pnas.1302212110 23959870 PMC3767519

[B56] Sparks D. L. Friedland R. Petanceska S. Schreurs B. G. Shi J. Perry G.. (2006). Trace copper levels in the drinking water, but not zinc or aluminum influence CNS Alzheimer-like pathology. J. Nutr. Health Aging 10, 247–254. 16886094 PMC3899576

[B57] Squitti R. (2012). Copper dysfunction in Alzheimer's disease: from meta-analysis of biochemical studies to new insight into genetics. J. Trace Elements Med. Biol. 26, 93–96. 10.1016/j.jtemb.2012.04.012 22565015

[B58] Squitti R. Faller P. Hureau C. Granzotto A. White A. R. Kepp K. P. (2021). Copper imbalance in Alzheimer's disease and its link with the amyloid hypothesis: towards a combined clinical, chemical, and genetic etiology. J. Alzheimers Dis. 83, 23–41. 10.3233/JAD-201556 34219710

[B59] Squitti R. Siotto M. Polimanti R. (2014). Low-copper diet as a preventive strategy for Alzheimer's disease. Neurobiol. Aging 35(Suppl. 2), S40–S50. 10.1016/j.neurobiolaging.2014.02.031 24913894

[B60] Strausak D. Mercer J. F. Dieter H. H. Stremmel W. Multhaup G. (2001). Copper in disorders with neurological symptoms: Alzheimer's, Menkes, and Wilson diseases. Brain Res. Bull. 55, 175–185. 10.1016/s0361-9230(01)00454-3 11470313

[B61] Suh S. W. Jensen K. B. Jensen M. S. Silva D. S. Kesslak P. J. Danscher G.. (2000). Histochemically-reactive zinc in amyloid plaques, angiopathy, and degenerating neurons of Alzheimer's diseased brains. Brain Res. 852, 274–278. 10.1016/s0006-8993(99)02096-x 10678753

[B62] Vural H. Demirin H. Kara Y. Eren I. Delibas N. (2010). Alterations of plasma magnesium, copper, zinc, iron and selenium concentrations and some related erythrocyte antioxidant enzyme activities in patients with Alzheimer's disease. J. Trace Elements Med. Biol. 24, 169–173. 10.1016/j.jtemb.2010.02.002 20569929

[B63] Watt N. T. Whitehouse I. J. Hooper N. M. (2010). The role of zinc in Alzheimer's disease. Int. J. Alzheimers Dis. 2011:971021. 10.4061/2011/971021 21197404 PMC3010690

[B64] Xu J. Church S. J. Patassini S. Begley P. Waldvogel H. J. Curtis M. A.. (2017). Evidence for widespread, severe brain copper deficiency in Alzheimer's dementia. Metallomics 9, 1106–1119. 10.1039/c7mt00074j 28654115

[B65] Yu J. Luo X. Xu H. Ma Q. Yuan J. Li X.. (2015). Identification of the key molecules involved in chronic copper exposure-aggravated memory impairment in transgenic mice of Alzheimer's disease using proteomic analysis. J. Alzheimers Dis. 44, 455–469. 10.3233/JAD-141776 25352456

